# Case report: Peripheral nerve stimulation relieves post-traumatic trigeminal neuropathic pain and secondary hemifacial dystonia

**DOI:** 10.3389/fneur.2023.1107571

**Published:** 2023-02-14

**Authors:** Junchi Li, Yongjie Li, Wei Shu

**Affiliations:** Department of Functional Neurosurgery, Xuanwu Hospital of Capital Medical University, Beijing, China

**Keywords:** post-traumatic neuropathic pain, trigeminal nerve, peripheral nerve stimulation (PNS), peripheral induced movement disorders, neuromodulation

## Abstract

Post-traumatic trigeminal neuropathic pain (PTNP) combined with secondary dystonia are rare sequelae of orofacial injury and often do not respond to conservative treatment. The consensus on treatment for both symptoms is yet to be standardized. This study reports the case of a 57-year-old male patient with left orbital trauma who developed PTNP immediately after the injury and secondary hemifacial dystonia 7 months thereafter. To treat his neuropathic pain, we performed peripheral nerve stimulation (PNS) using a percutaneously implanted electrode to the ipsilateral supraorbital notch along the brow arch, which instantly resolved the patient's pain and dystonia. PTNP was relieved in a satisfactory manner until 18 months after the surgery, despite a gradual recurrence of the dystonia since 6 months after the surgery. To the best of our knowledge, this is the first reported case of PNS used for the treatment of PTNP combined with dystonia. This case report highlights the potential benefits of PNS in relieving neuropathic pain and dystonia and discusses the underlying therapeutic mechanism. Moreover, this study suggests that secondary dystonia occurs due to the uncoordinated integration of afferent sensory and efferent motor information. The findings of the present study indicate that PNS should be considered for patients with PTNP following the failure of conservative treatment. Secondary hemifacial dystonia may benefit from PNS upon further research and long-term assessment.

## Introduction

Post-traumatic trigeminal neuropathic pain (PTNP) is defined as chronic pain over 3 months in the trigeminal dermatomal region caused by trauma or iatrogenic injury to the trigeminal nerve. Approximately 3–15% of people with trigeminal nerve injury develop neuropathic pain (1–3). Consistent or paroxysmal symptoms, including burning, tingling, or shooting pain, are present in the orofacial region that is innervated by the injured trigeminal nerve branch (4). Additionally, in rare cases, segmental dystonia can occur following peripheral nerve injury, causing suffering and social embarrassment to the patient (5). Both these conditions reduce the patient's quality of life and lead to long-term burdens posed by medical care (3, 6, 7). Despite the disastrous consequences, effective therapies for the treatment of these conditions are limited, and the pathophysiology remains unclear.

Herein, we report the case of a patient with PTNP who developed segmental dystonia in the ipsilateral orofacial region. To the best of our knowledge, this is the first study to report on PTNP combined with secondary hemifacial dystonia. Furthermore, to date, no study has reported on the use of peripheral nerve stimulation (PNS) to relieve hemifacial dystonia. This study also discusses the underlying mechanism of neuropathic pain combined with dystonia, as both are modulated by PNS.

## Case presentation

**Figure 1:**
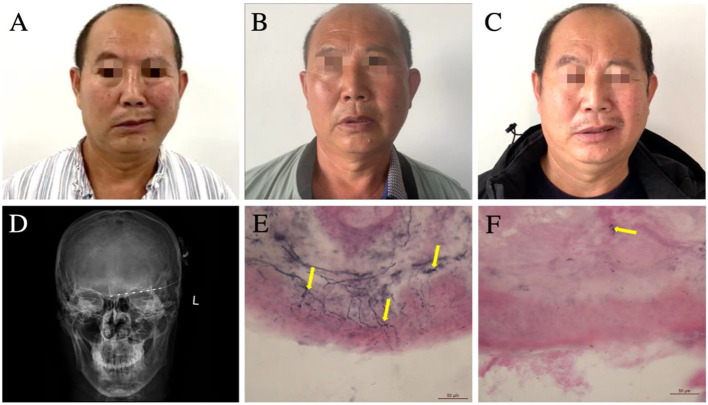
**(A–C)** Changes in the patient's hemifacial and cervical dystonia over time. **(A)** Pre-implant surgery, the dystonia on the left side of the patient's face is seen, manifesting as an involuntary contraction of the left hemifacial. **(B)** Satisfactory relief of the dystonia is still evident at 6 months after PNS surgery. **(C)** Involuntary contraction of the left-sided facial muscles reappeared at 12 months after PNS surgery. **(D)** Anteroposterior radiograph of the cranium demonstrating the placement of the supraorbital PNS-stimulating electrodes. **(E, F)** Bilateral forehead skin biopsy using immunolabeled protein gene product 9.5 demonstrating markedly lower epidermal neurite (yellow arrows) density in the left forehead **(F)** vs. that in the right forehead **(E)**. PNS, peripheral nerve stimulation.

A 57-year-old male patient was admitted to our hospital with left forehead pain for 11 months. He had been stabbed by a metal rod in the left orbital cave, close to the supraorbital notch. Computed tomography did not show any intracranial injury or hemorrhage, and the wound was debrided. Following the healing of the wound, he experienced persistent, severe, burning pain in the left forehead, and this patient was unresponsive to various medications (including gabapentin, tramadol, and carbamazepine) for the pain. Seven months after the injury, he developed segmental dystonia on the left side of the face, presenting as a constant involuntary muscle contraction. The hemifacial dystonia had persisted for 4 months at the time of admission (Figure 1A). Physical examination revealed that the skin over the left forehead had become thin, with red discolouration and had markedly decreased sensation. Sustained muscles contracted were observed on his left cheek. Platysma muscle spasm also appeared, leading to the neck that appeared slightly tilted to the left. However, cervical dystonia was neither observed nor subjectively reported by the patient. The patient was able to rotate his neck freely, and the muscle tone of the sternocleidomastoid muscle was normal. Based on these findings, we concluded that dystonia only affected the platysma muscle innervated by the cervical branch of the facial nerve and did not involve deep neck muscles. The latency of the blink reflex on the left side of the face was marginally extended, indicating partial supraorbital nerve damage. To assess intra-epidermal nerve fibers, skin biopsy with a 3-mm punch instrument was performed symmetrically on the bilateral forehead according to the pain site, ~2 cm above the midpoint of the eyebrows. Labeled with protein gene product 9.5, the immunohistochemistry of the left forehead revealed severe denervation of intra-epidermal and dermal nerve fibers (Figures 1E, F). Based on the aforementioned results, neuropathic pain caused by trigeminal nerve injury was considered. The primary goal of therapy is to relieve the pain. First, to identify the origin of neuropathic pain and the cause of dystonia, we performed a supraorbital notch nerve block using 1% lidocaine, which alleviated the pain and hemifacial dystonia for 1 day. As a result, we confirm the critical pathogenic site and the target of neurostimulation. Second, a PNS electrode (Model 3777, Medtronic, Minneapolis, MN, USA) was implanted percutaneously from the left temporal region to the ipsilateral supraorbital notch, along the brow arch (Figure 1D). During the subsequent 7-day trial, the patient experienced satisfactory relief of both the pain and facial dystonia. Finally, a permanent pulse generator was implanted in the left chest below the clavicle bone. The implanted percutaneous lead was connected to an extension wire tunneled behind the left ear and down the neck to reach the internal pulse generator. After the operation, the neck moved as freely as during pre-operation, without any sign of neck muscle injury. At discharge, the visual analog scale score had decreased from 9 to 1. The hemifacial dystonia alleviates from a baseline score of 23 to 5, as measured using the Burke–Fahn–Marsden Dystonia Rating Scale (BFMDRS) (Table 1). The PNS impulse generator was activated by the patient himself only 3–4 times per day manually as the therapeutic effect lasted 4–6 h following the 1-h stimulation (1–, 3–, 8+; 220 ms; 40 Hz; 1.5 mA).

**Table 1:** Burke–Fahn–Marsden Dystonia Rating Scale scores of the patient with hemifacial and cervical dystonia.

**Movement and disability scale score**	**Baseline**	**PNS-6 months**	**PNS-12 months**	**Botulism injection**
Eyes (0–8)	6	0	6	0
Mouth (0–8)	8	2	8	0
Speech and swallowing (0–16)	1	0	1	0
Neck and trunk (0–24)	6	3	6	6
Speech (0–4)	1	0	1	0
Writing (0–4)	0	0	0	0
Feeding (0–4)	0	0	0	0
Eating and swallowing (0–4)	1	0	1	0
Hygiene (0–4)	0	0	0	0
Dressing (0–4)	0	0	0	0
Walking (0–6)	0	0	0	0
Total	23	5	23	6

Baseline, pre-peripheral nerve stimulation surgery; PNS-6 months and PNS-12 months, at 6- and 12-month post-peripheral nerve stimulation surgery, respectively.

Six months after the surgery, the patient returned to our department for a follow-up. He was satisfied with the efficacy of PNS (Figure 1B). However, the dystonia recurred gradually at 12 months after the surgery, and the pain-relieving effect persisted; however, the facial dystonia had worsened (Figure 1C; Table 1). We injected botulinum toxin to alleviate the hemifacial dystonia with a good outcome, and the BFMDRS score decreased to 6 at 18 months after the surgery (Table 1).

## Discussion

We report the rare case of peripheral PTNP and segmental dystonia, which were simultaneously relieved by PNS. To the best of our knowledge, no previous study in the literature has reported on the use of PNS for the simultaneous relief of neuropathic pain and dystonia. Compared with the longlasting analgesic effect, the dystonia recurred gradually after 6 months. This suggests that, apart from pain, the abnormal facial sensation was the crucial factor for secondary dystonia. Herein, we describe the underlying mechanism.

Post-traumatic trigeminal neuropathic pain may result from an abnormal regeneration of the trigeminal nerve after injury. During the repair of the injured nerve, regenerated neural tissues, including those of axons and Schwann cells, eliminate inflammation and regulate the hyperexcitability of injured nerve axons. If nerve regeneration fails, the chronic neuropathic pain worsens (8). In our case study, the results of periorbital skin biopsy showed a significant decrease in the density of nerve fibers on the affected side, indicating that regeneration of damaged axons failed to reinnervate the target region. Due to disturbances in this restoration process, paraesthesia and pain were experienced by the patient. Similar pathological changes, including disintegrated swollen axons and a sparse density of nerve fibers, have been found in small-fiber polyneuropathy, also contributing to neuropathic pain (9). To our knowledge, this is the first report on skin pathology with regard to post-traumatic trigeminal nerve injury.

Post-traumatic trigeminal neuropathic pain is challenging for physicians as it is often refractory to analgesics (2). Traditional surgical treatments include radiofrequency ablation, neurotomy, and neurolysis. However, their effectiveness is limited and temporary. Additionally, operation resulting in the destruction of the ophthalmic nerve may cause serious complications, such as conjunctivitis and corneal ulcer. Thus, neuromodulation therapy may be preferred for the treatment of PTNP, owing to its minimally invasive, reversible, and modifiable characteristics (10).

Peripheral nerve stimulation is a neuromodulation technique that is used to manage neuropathic pain following pharmacological analgesic failure. Studies on PNS, which involved post-traumatic, post-herpes zoster, and idiopathic painful trigeminal neuropathy, showed satisfactory long-term outcomes in 70–86.4% of patients (10–12). Variability in the outcomes of PNS may be related to aetiological factors. PTNP is likely to yield satisfactory outcomes after PNS (11). In our case, incomplete ophthalmic nerve injury resulted in chronic deafferent neuropathic pain. Consequently, PNS was considered after the failure of pharmacological analgesia. A supraorbital notch nerve block with lidocaine was performed to identify the origin of pain, which was also a predictive factor for the efficacy of PNS. The electrode lead was then placed parallel to the brow arch while covering the supraorbital notch to stimulate the supraorbital and supratrochlear nerves. PNS-stimulated myelinated Aβ fibres activate inhibitory gamma-aminobutyric acid-ergic interneurons, which suppress the transmission of nociceptive information from C and A delta (Aδ) fibers to the central nervous system (CNS), resulting in decreased pain and reserved or strengthened Aβ fiber input. In the patient mentioned in this report, PNS generated paraesthesia over the affected area and effectively relieved neuropathic pain.

**Figure 2:**
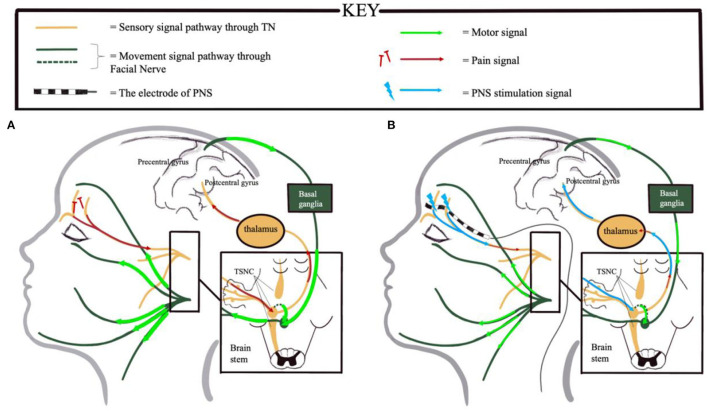
Illustration of the potential mechanism by which electric stimulation *via* PNS may simultaneously relieve neuropathic pain and dystonia in PTNP by influencing sensorimotor integration processes. **(A)** PTNP arises in the region of the forehead innervated by the supraorbital and supratrochlear nerves. Trigeminal sensory afferent neurons convey amplified nociceptive signals (red arrows) to the ipsilateral TSNC. Next, the cortical and subcortical structures integrate the sensory information and transmit aberrant movement signals through the facial nucleus and motor efferent neurons, though occasionally, integrated signals are projected directly from the TSNC to the facial nucleus (13) (indicated by dotted green lines). **(B)** PNS modulates the pain signals (blue arrows and fine red arrows) and downregulates the efferent motor information (fine green arrows). PNS, peripheral nerve stimulation; PTNP, post-traumatic trigeminal neuropathic pain; TSNC, trigeminal sensory nuclear complex.

Sensorimotor network incoordination is central to the pathogenesis of post-traumatic facial dystonia, affected by PTNP and CNS reorganization. Facial skin mechanoreceptors of the trigeminal nerve collect sensory information, which is transferred to several structures in the CNS, such as the trigeminal sensory nuclear complex, the thalamus, the basal ganglia, and the cortex (13). These structures functionally connect with motor nuclei to regulate facial muscle movement. Abnormal integration of the trigeminal nerve–CNS–facial nerve pathway may result in mismatched signals and produce involuntary facial muscle movement. The temporary elimination of PTNP by the supraorbital nerve block and the later immediate relief of involuntary facial muscle contraction by PNS indicated that efferent motor activity was regulated by pain signals. CNS reorganization caused by chronic pain or hyperaesthesia may be another key factor in the development of dystonia, as indicated by the 7-month delay in the appearance of ipsilateral involuntary facial movements after trauma (5, 14). The constant input of abnormal sensations from the injured trigeminal nerve promotes abnormal cortex or subcortex structure remodeling and leads to inaccurate sensorimotor integration processes, which result in exporting of faulty signals to the facial muscles (Figure 2A) (15). Tic douloureux, a hemifacial spasm caused by trigeminal neuralgia, may also share this mechanism.

In addition to the role of PNS in alleviating pain, the mechanism by which PNS relieves facial dystonia remains unclear. Sensory inputs, including pain, touch, and proprioception, are modulated by PNS, which may be a crucial factor. We hypothesize that PNS modulates the abnormal integration of sensory and motor information to relieve dystonia (Figure 2B), similar to traditional alleviating maneuvers (sensory trick maneuvers) (15, 16). Unfortunately, the patient's dystonia started gradually recurring 6 months after surgery despite adequate pain relief, which may have been influenced by persistent abnormal CNS plasticity. We hypothesized that, although the pain had been controlled effectively, disordered sensory input from the injured trigeminal nerve afferents, such as aberrant feedback from denervation of the frontalis muscle, may have continued to cause further CNS reorganization. This would have been difficult to reverse using PNS, and thus, the dystonia recurred. Nonetheless, this study supports the potential of modulating sensory inputs *via* trigeminal afferents as a treatment intervention for facial dystonia (13).

Recently, botulinum toxin has been used to alleviate pain and dystonia simultaneously, particularly musculoskeletal pain and lower back pain (17). In addition to reducing abnormal muscle contraction by inhibiting the release of acetylcholine at the neuromuscular junction, this toxin may relieve neuropathic pain through other mechanisms, such as improving blood supply (18), reducing pain-related mediators, and inhibiting central perception (17). In the patient mentioned in our study, botulinum toxin successfully inhibited the dystonia during the later period of treatment but failed to relieve pain, potentially because this patient's neuropathic pain both preceded the dystonia and extended beyond the area of severe muscle spasm.

## Conclusion

We report a rare case of trigeminal nerve injury, which involved neuropathic pain combined with segmental dystonia. PTNP may be an initiating factor for dystonia. PNS should be considered as an alternative method for alleviating neuropathic pain if pharmacological treatment fails. Moreover, PNS may be helpful to modulate sensorimotor information that is capable of temporarily switching off dystonic activity. Thus, further research into the underlying mechanism is required.

## Data availability statement

The original contributions presented in the study are included in the article/supplementary material, further inquiries can be directed to the corresponding author.

## Ethics statement

Written informed consent was obtained from the individual(s) for the publication of any potentially identifiable images or data included in this article.

## Author contributions

JL and WS reviewed the literature and contributed to manuscript drafting. YL and WS were responsible for revision of the manuscript for important intellectual content. All authors issued final approval for the version to be submitted.
